# Endovascular Treatment for Pseudoaneurysms After the Surgical Repair of Aortic Coarctation

**DOI:** 10.7759/cureus.8978

**Published:** 2020-07-03

**Authors:** Wail Alkashkari, Faisal Al-Husayni, Mawaddah Alfouti, Rahaf Alsofyani, Sarah Alfawaz

**Affiliations:** 1 Cardiology, King Faisal Cardiac Center, King Abdulaziz Medical City, Ministry of National Guard Health Affairs, Jeddah, SAU; 2 Cardiology, King Abdullah International Medical Research Center, King Saud Bin Abdulaziz University for Health Sciences, Jeddah, SAU; 3 Internal Medicine, National Guard Hospital, King Abdulaziz Medical City, Jeddah, SAU

**Keywords:** aortic pseudoaneurysm, aortic coarctation, endovascular, aortic stenting, transcatheter repair, endograft implantation

## Abstract

In this report, we describe the case of a 28-year-old male who presented to our hospital with shortness of breath and sudden, severe central chest pain that radiated across his chest and back. The patient had a history of coarctation of the aorta (CoA) repair using Dacron patch aortoplasty at the age of 10 years, and he had been lost to clinical follow-up. A chest X-ray (CXR) revealed the widening of the upper mediastinum. He underwent emergency CT angiography, which demonstrated extensive mediastinal hematoma and contrast leaking from a 4x12 cm complex pseudoaneurysm of the proximal thoracic descending aorta. After the heart-team meeting, the transcatheter approach was deemed more feasible and safer than a surgical approach. The patient was taken to cardiac catheterization laboratory and, under general anaesthesia, we successfully implanted a tapered (28 mm - 26 mm) x 150 mm Valiant Thoracic Stent Graft with the Captivia Delivery System (Medtronic Vascular, Santa Rosa, CA). In this case, we demonstrated the feasibility and safety of using a stent graft to treat late surgical complications after CoA repair, which are not uncommon.

## Introduction

Coarctation of the aorta (CoA) is the sixth most common congenital heart disease (CHD), accounting for 4-8% of all CHD. It occurs in four out of 1,000 live births and has a male predominance [1]. Surgical repair of CoA has been the standard treatment in infants and adolescents to prevent either the early or late consequences of obstruction, proximal hypertension, and distal hypoperfusion [2]. Several surgical techniques for CoA repair have been applied traditionally, which include resection with end-to-end anastomosis, extended end-to-end anastomosis, prosthetic patch aortoplasty, subclavian artery flap aortoplasty, interposition tube graft, and extra-anatomical aortic bypass [2]. Currently, adults presenting late are generally treated with balloon-expandable stents, with excellent immediate and long-term outcomes [1,3]. Irrespective of the kind of surgical technique used initially, late post-repair complications are not uncommon, and often occur decades later. Such complications include hypertension, re-CoA, and aneurysm/pseudoaneurysm formation. Hence, it is important that patients are kept under lifetime surveillance, which includes regular cross-sectional imaging, blood pressure monitoring, and risk factor modification. Many patients have been discharged or lost to follow-up before its importance was appreciated. Some patients may present with a symptomatic or apparently incidental but serious complication years after the first repair. Although any surgical technique can lead to late pseudoaneurysm formation at the anastomotic site (around 10% of cases overall), longer-term follow-up suggests that there is a particular problem with patch aortoplasty. Incidence of late pseudoaneurysm associated with this technique may reach as high as 38%, although lower figures have also been recorded, and our current understanding might have been affected by the large number of patients who are no longer under follow-up. The average time to aneurysm formation has been reported to be up to 12 years after the initial repair, with a progressive increase in prevalence over time. As such, we can anticipate a growing problem in the future. Emergency presentation is not uncommon (with rupture being associated with 7% mortality), as in the complex case we present in this report [2].

## Case presentation

**Figure 1:**
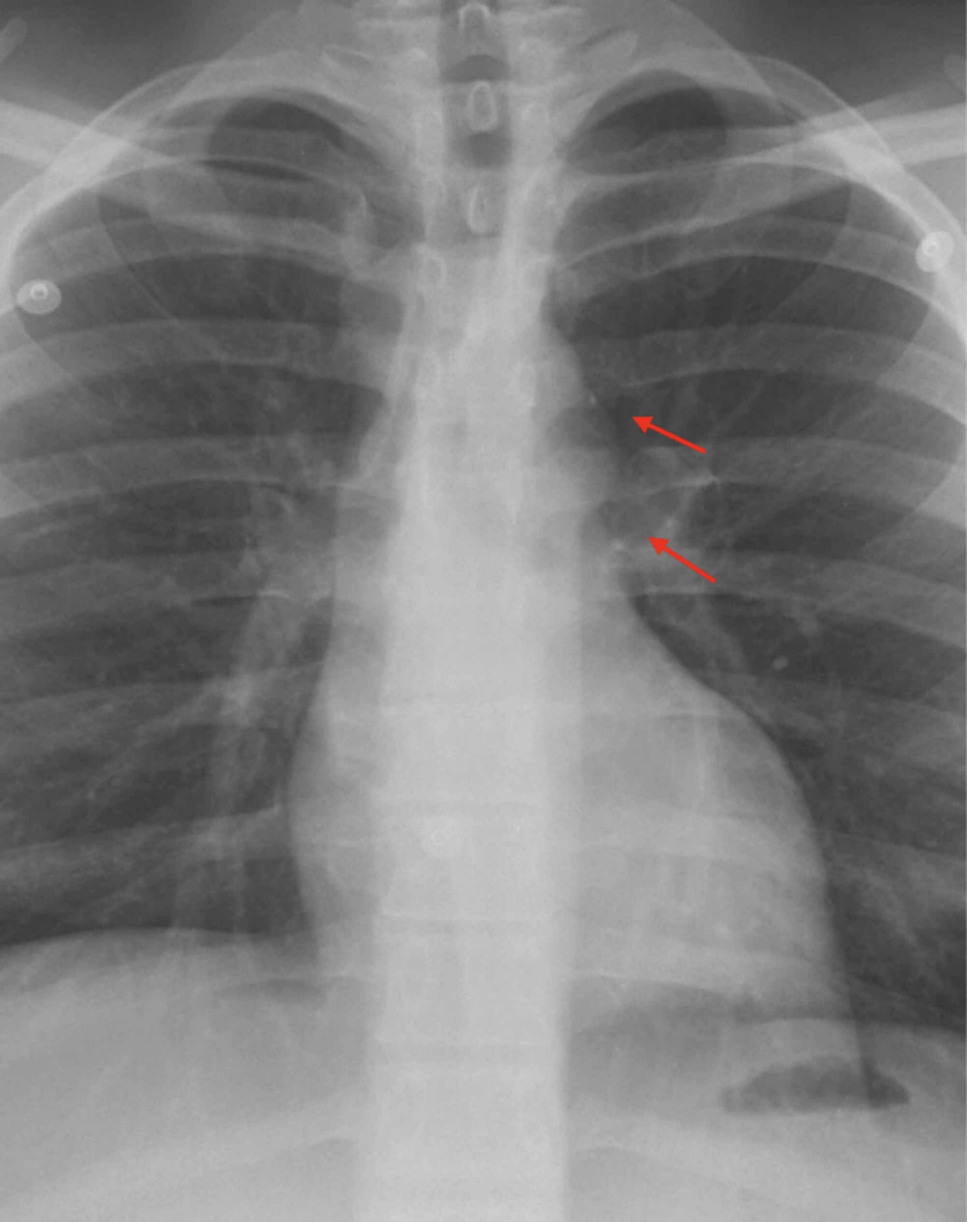
Chest X-ray revealing wide mediastinum (red arrows)

A 28-year-old man, with a history of CoA repair using Dacron patch aortoplasty at the age of 10 years, presented with shortness of breath and sudden, severe central chest pain that radiated across his chest and back. The patient had been lost to clinical follow-up over the last 15 years. He was conscious, hypotensive (90/60 mmHg on both arms), and in distress with tachycardia (120 BPM) and relatively weak pedal pulses with cold distal extremities. A chest X-ray (CXR) demonstrated the widening of the upper mediastinum (Figure 1).

**Figure 2:**
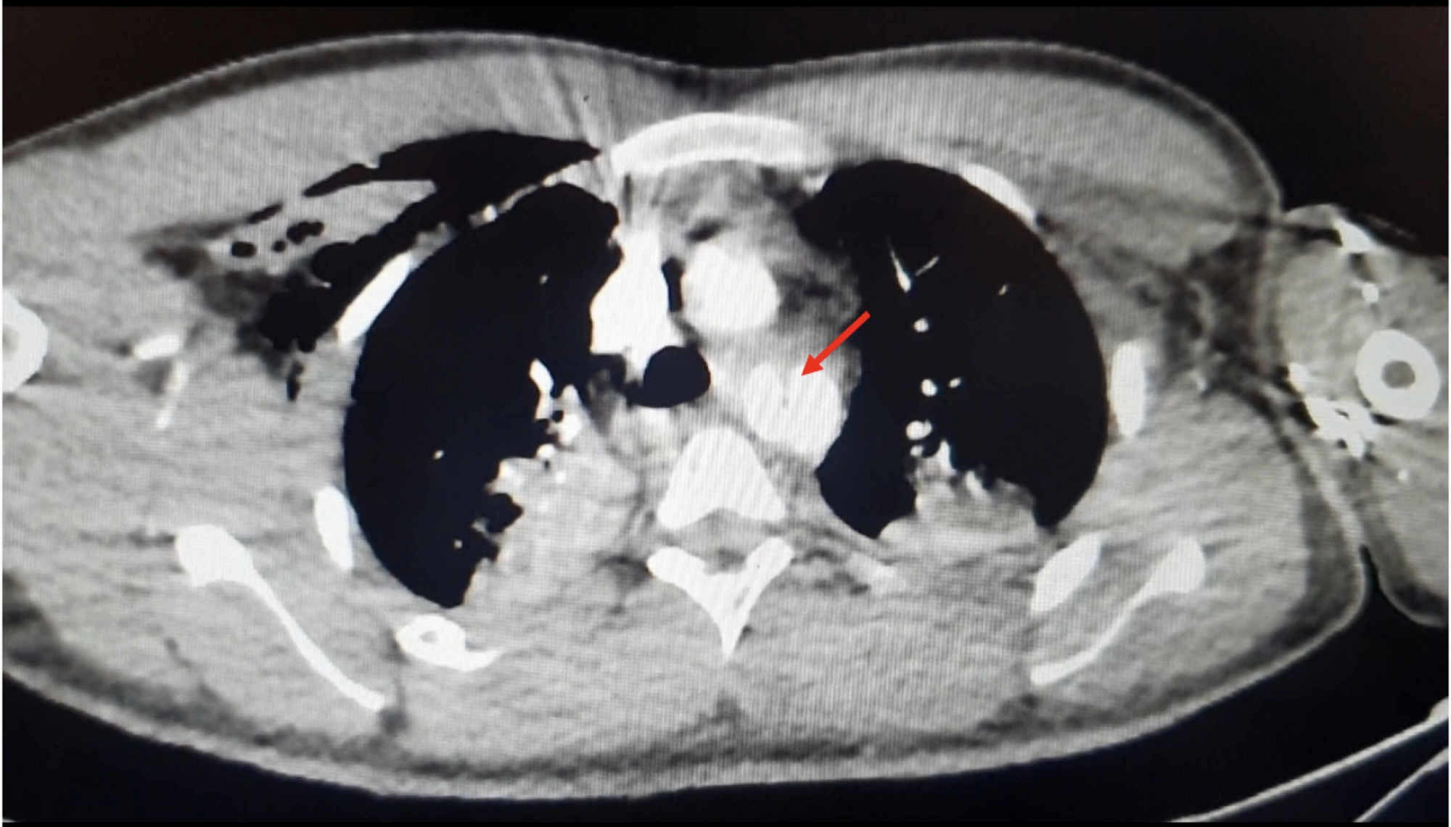
CT angiography of the patient (non-ECG-gated) The image demonstrates intimal disruption starting about 2 cm distal to the origin of the left subclavian artery (red arrow) CT: computed tomography; ECG: electrocardiogram

He underwent emergency CT angiography, which demonstrated extensive mediastinal hematoma and contrast leaking from a 4x12 cm complex pseudoaneurysm of the proximal thoracic descending aorta (Figure 2).

**Figure 3:**
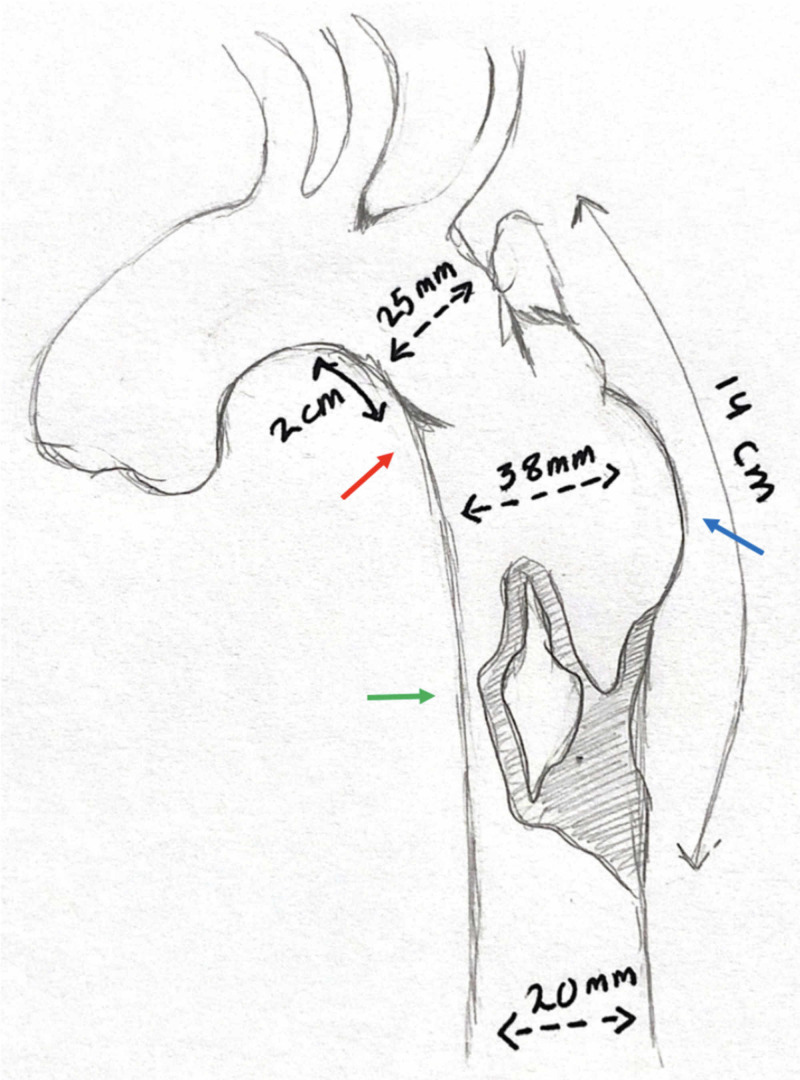
A sketch representing the CT angiography findings The sketch shows intimal flap at the site of disruption (red arrow), large pseudoaneurysm (blue arrow), and aortic true lumen compression (green arrow). We usually do such sketches to plan our approach (roadmap) CT: computed tomography

The intimal disruption started about 2 cm distal to the origin of the left subclavian artery (LSCA), which was probably the site of the patch repair. The aortic diameter just prior to the disruption was 2.5 cm, and the normal distal thoracic aortic diameter was 2 cm. Of note, there was compression to the distal aorta by the pseudoaneurysm, leading to significant narrowing, which explained the weak distal pulses and cold extremities. After the heart-team discussion, the decision was made to proceed with transcatheter endograft implantation. The transcatheter approach was deemed more feasible and safer than a surgical one, especially in the presence of a good landing zone for endograft placement (away from the LSCA) (Figure 3).

**Figure 4:**
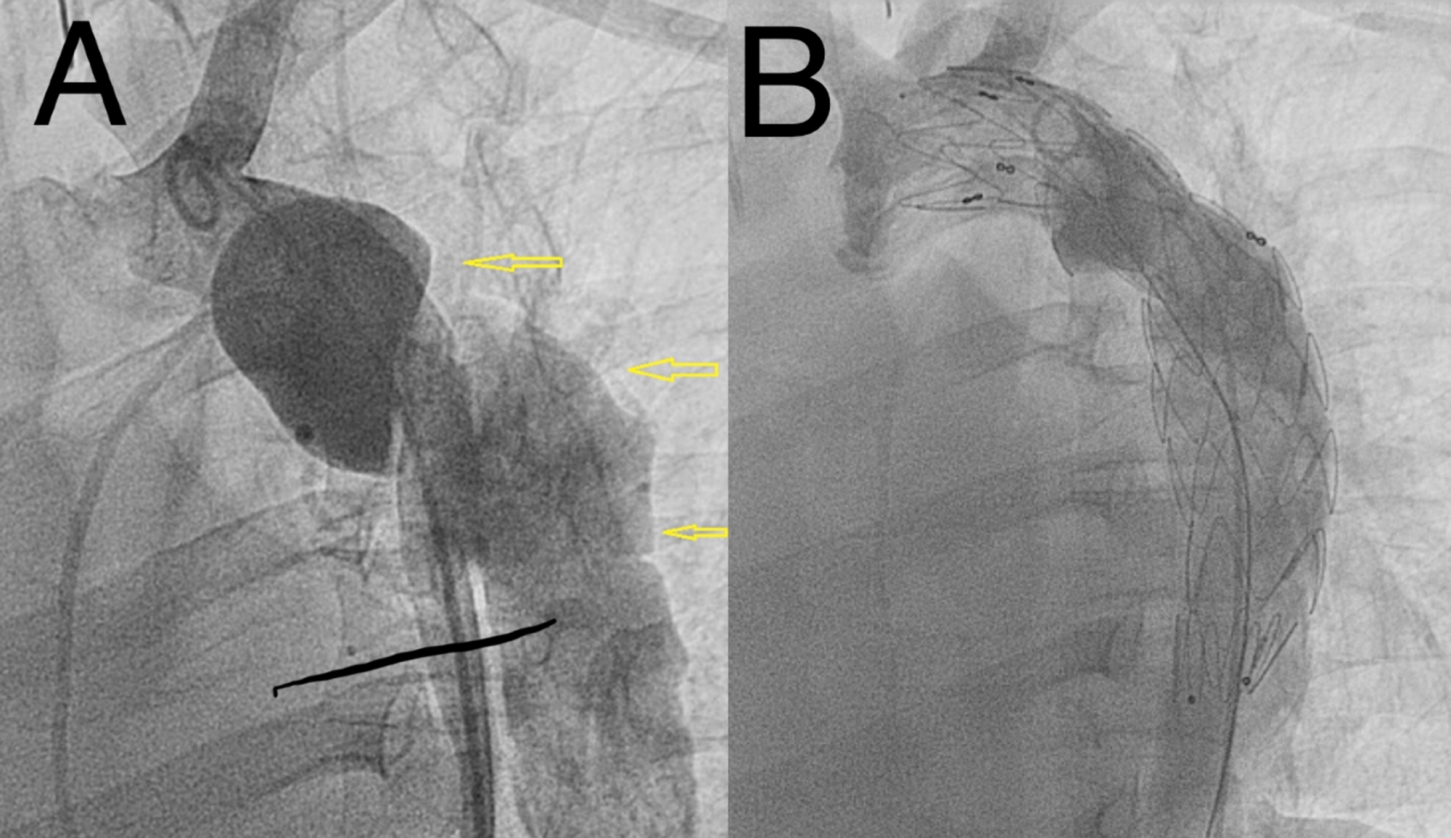
Aortic angiography findings A: aortic angiography demonstrating aortic intimal disruption and large complex pseudoaneurysm (yellow arrows). B: aortic angiography revealing successful endograft stent implantation with no evidence of endoleak and a patent left subclavian artery

The patient was taken to cardiac catheterization laboratory and, under general anesthesia, we implanted a tapered (28 mm - 26 mm) x 150 mm Valiant Thoracic Stent Graft with the Captivia Delivery System (Medtronic Vascular, Santa Rosa, CA). The endograft was implanted successfully with complete exclusion of the pseudoaneurysm and excellent flow distally (Figure 4).

The access site (right femoral artery) was secured successfully with two Perclose ProGlide™ devices (Abbott Laboratories, Abbott Park, IL). The patient did very well and was discharged home after three days. A follow-up CT angiography revealed a widely patent stent graft with no endoleaks.

## Discussion

The surgical repair of CoA is generally associated with good long-term survival, with rates approaching 90% 25-year survival in those operated on in childhood. However, late complications are still common [4]. Redo open surgery for late surgical complications is challenging and associated with significant mortality and morbidity (including damage of the recurrent laryngeal nerve, paraplegia, and bleeding). Late pseudoaneurysms are not uncommon and should not be left untreated, as rupture has been described even in small pseudoaneurysms [4]. Knyshov et al. have reported a rupture rate of 100% at 15 years post-procedure for these pseudoaneurysms [5].

Recently, endovascular approaches have been gaining favor in selected cases for reducing complications related to redo open surgery. Even though the reported series have been small, they show very encouraging results. Reports and studies have shown a procedural success rate of up to 100% [6]. However, long-term data relating to stent-graft performance in these young patients are scarce. The technical difficulty of treating these patients includes proximal fixation zone in relation to the extension of the pseudoaneurysm, the dimension of the access arteries, and the presence of hypoplastic aortic arch. Potential complications are similar to those caused by balloon angioplasty or stent placement for re-CoA, which includes aortic wall injury and access-site complications [2]. Endoleaks can occur due to suboptimal endograft apposition to the aortic wall, especially in cases of challenging anatomy [6]. Another devastating complication that can occur in up to 10% of cases after stent-graft implantation (especially for long grafts of >20 cm) is spinal cord ischemia, leading to paraparesis or paraplegia. Proper evaluation and sizing are crucial to avoid such complications [7]. In our case, there was a good proximal (away from the LSCA) and distal landing zone and a relatively short complex lesion, which enabled us to implant the graft stent successfully without jailing the LSCA and causing damage to the spinal artery.

## Conclusions

Pseudoaneurysm after the surgical repair of CoA is likely to be a serious problem in the future. Redo open surgery for late surgical complications is challenging and associated with significant mortality and morbidity. Endovascular treatment is a viable alternative to surgical reoperation due to its lower morbidity and mortality. However, more data from long-term monitoring are needed to determine their effectiveness and to detect late complications.
